# Segmentation of the subcuticular fat body in *Apis mellifera* females with different reproductive potentials

**DOI:** 10.1038/s41598-021-93357-8

**Published:** 2021-07-06

**Authors:** Aneta Strachecka, Krzysztof Olszewski, Karolina Kuszewska, Jacek Chobotow, Łukasz Wójcik, Jerzy Paleolog, Michał Woyciechowski

**Affiliations:** 1 Department of Zoology and Animal Ecology, University of Life Sciences in Lublin, Lublin, Poland; 2 Institute of Biological Basis of Animal Production, University of Life Sciences in Lublin, Lublin, Poland; 3 Institute of Environmental Sciences, Jagiellonian University, Kraków, Poland; 4 Faculty of Biology and Biotechnology, Maria Curie-Sklodowska University, Lublin, Poland

**Keywords:** Animal physiology, Entomology

## Abstract

Evolution has created different castes of females in eusocial haplodiploids. The difference between them lies in their functions and vulnerability but above all in their reproductive potentials. Honeybee queens are highly fertile. On the other hand, the workers are facultatively sterile. However, rebel workers, i.e. workers that develop in a queenless colony, reproduce more often than normal workers. As a result, the fat body of these bees, which apart from acting as the energy reserve, is also the site of numerous metabolic processes, had to specialize in different functions perfected over millions of years of eusocial evolution. Assuming that the variety of functions manifests itself in the pleomorphic structure of the fat body cells, we predicted that also different parts of the fat body, e.g. from different segments of the abdomen, contain different sets of cells. Such differences could be expected between queens, rebels and normal workers, i.e. females with dramatically different reproductive potentials. We confirmed all these expectations. Although all bees had the same types of cells, their proportion and segmental character corresponded with the caste reproductive potential and physiological characteristics shaped in the evolutionary process. The females with an increased reproductive potential were characterized by the presence of oenocytes in the third tergite and high concentrations of compounds responsible for energy reserves, like glucose, glycogen and triglycerides. Queens had very large trophocytes, especially in the third tergite. Only in workers did we observe intercellular spaces in all the segments of the fat body, as well as high protein concentrations—especially in the sternite. As expected, the rebels combined many features of the queens and normal workers, what with other findings can help understand the ways that led to the origin of different castes in females of eusocial Hymenoptera.

## Introduction

The switch from a predatory to a herbivorous lifestyle was a key to the tremendous diversification of bees in the Cretaceous^1^. Such evolution by natural selection involves DNA sequence mutations and chromosomal alterations that result in heritable phenotypic variation, e.g. in eusocial bees^2–4^. Additionally, eusociality in insects provides an example of convergent phenotypic innovation^5,6^. The evolutionary success of the social-life strategy in Hymenoptera is connected with a marked division of labour between female members of the society^7–9^. In the advanced eusocial insects the reproductive role is limited to one or a few highly specialised queens supported by a mostly sterile worker caste. This segregates investment in reproduction and colony growth and maintenance between different individuals, which increases the total reproductive output of the group of kin^10–13^. Consequently, reproductive division of labour shaped the queen-*versus*-worker dimorphism involving plasticity in body size and ovary development^14^. Workers, however, usually retain the ability to activate their ovaries and facultatively lay unfertilised, male-destined eggs, thereby inciting possible conflict over male parentage^15,16^. Most honeybee workers are sterile but there are situations in which rebels, i.e. workers with a high reproductive potential, appear^17^. The rebels develop immediately after swarming when the old queen has flown away with a swarm and the new one is still in the pupa stage. The absence of an active queen and its mandibular pheromones in larval food affects the development of this sub-caste^18^. Compared with normal workers, the rebels have more ovarioles in the ovaries, less developed hypopharyngeal glands producing the larval food, while their mandibular glands, which produce queen mandibular pheromones in queens, are larger^19,20^.

Ovarian signalling in honeybee workers is related to social behaviour, endocrine sensitivity, and longevity^21–23^. This is consistent with an evolutionarily conserved link between fertility and life span^24–26^. Such ovarian factors as the ovary size, ovarian activity, and yolk protein levels result from the corpora allata activity that regulates juvenile hormone (JH) titers in workers^22^. The endocrine integration of worker life-history progression is governed by the corpora allata—ovarian axis^27^. This axis is associated with the fat body (corpus adiposum; analogous to vertebrate adipose and liver tissues). In honeybee workers, the fat body produces vitellogenin, which transcribes mRNA for insulin-like peptides and regulates JH titers, and therefore, worker behaviour^27–30^.

The insect fat body is the central storage depot for nutrients, which plays an essential role in energy storage and utilization. In addition, it is a tissue of great biosynthetic and metabolic activity^31–33^. It is mainly involved in the hormonal control of the storage and mobilization of energy reserves (fat and glycogen) that are crucial for the reproductive anabolic processes and for the group overwintering capabilities^34–36^. Moreover, fat bodies accumulate toxic and reserve compounds (e.g. urea, uric acid)^37^ and protect against oxidative stress, enhancing the apian resistance systems^38–41^. Roma et al.^42^ suggest that a few modifications had occurred during the evolution of the insects in the cells of the fat body, which resulted in the functional specialization of the individuals. Ruvolo and Cruz-Landim^43^ confirmed that each of the castes has its own specificity in terms of cell size and place of occurrence in the body (e.g. head, thorax, etc.). An example may be the trophocytes of the reproductive castes of Attini ants which, in comparison with the workers, possess higher amounts of cytoplasmic vacuoles containing mainly lipids and proteins, which suggests that these compounds are used during the processes involving oogenesis and vitellogenesis^44,45^. In turn, Roma et al.^46^ think that large amounts of polysaccharides in the trophocytes of *Mycetarotes parallelus* and *Atta laevigata* workers are used as an energy source, especially by the muscle system during the foraging activities. On the basis of this information, it can be expected that the fat body cells of the worker/reproductive castes/sub-caste will contain the same components (e.g. nuclei, vacuoles, and the protein and lipid stores), but will retain original morphological and physiological characteristics shaped in the evolutionary process.

Based on the above, the aim of this study was to identify the similarities and differences in the fat body morphology of honeybee queens, normal workers and rebels. At this point, another question arises: does the entire subcuticular fat body function in the same way or does its tissue differ in other segments, mainly morphologically and functionally, from the rest of the sites under the cuticle layer on the abdomen? Therefore, the second aim was to answer the above question. The fat body has been characterized as the visceral part which surrounds the internal organs, and as the subcuticle element which, thanks to the loose arrangement of cells and being washed by the hemolymph, ensures metabolite exchange^28,43,47,48^. We applied a new approach to analyzing the subcuticular part of the fat body, not as a whole comprised between the 2nd and 7th abdominal segments, but as separate sets of cells in individual segments. Therefore, we dissected cells of the subcuticular fat body from different places of the abdomen.

## Results

To confirm that females we had breaded belonged to the queen, rebel and normal worker castes, we examined their ovarian tubes. The highest numbers of the tubes were found in the queens. On average, the rebels had 7.3 more ovarian tubules than the normal workers (Supplementary Fig. S1). This result allowed us to continue research and compare the fat bodies in the rebels, queens and normal workers.

### Morphology of the fat body

**Figure 1:**
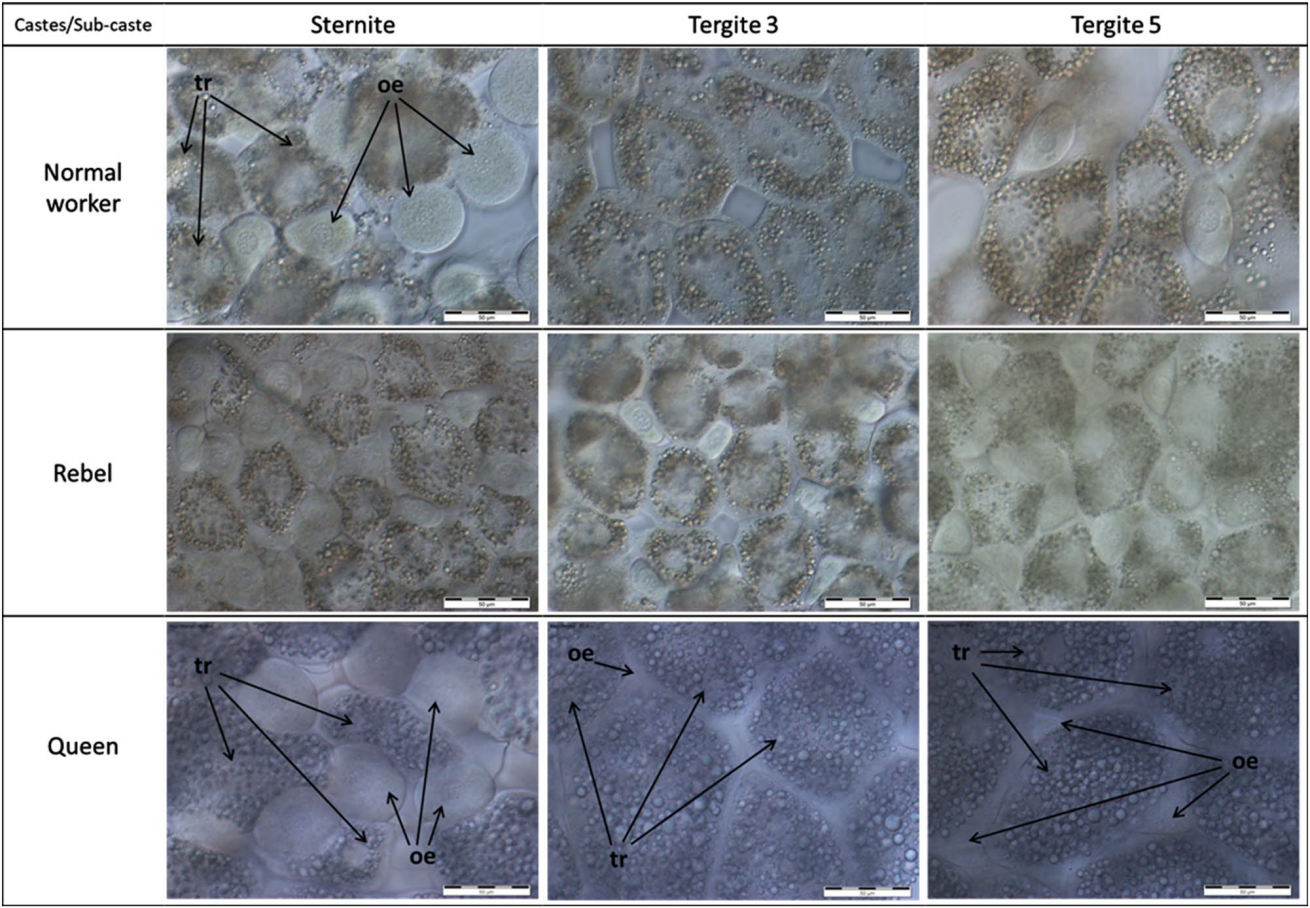
Histological features of fat body cells in *Apis mellifera* females. The fat bodies were analysed in the sternite and the third and the fifth tergite. Scale bar—50 μm; tr—trophocytes; oe—oenocytes; Tergite 3—the third tergite; Tergite 5—the fifth tergite.

**Figure 2:**
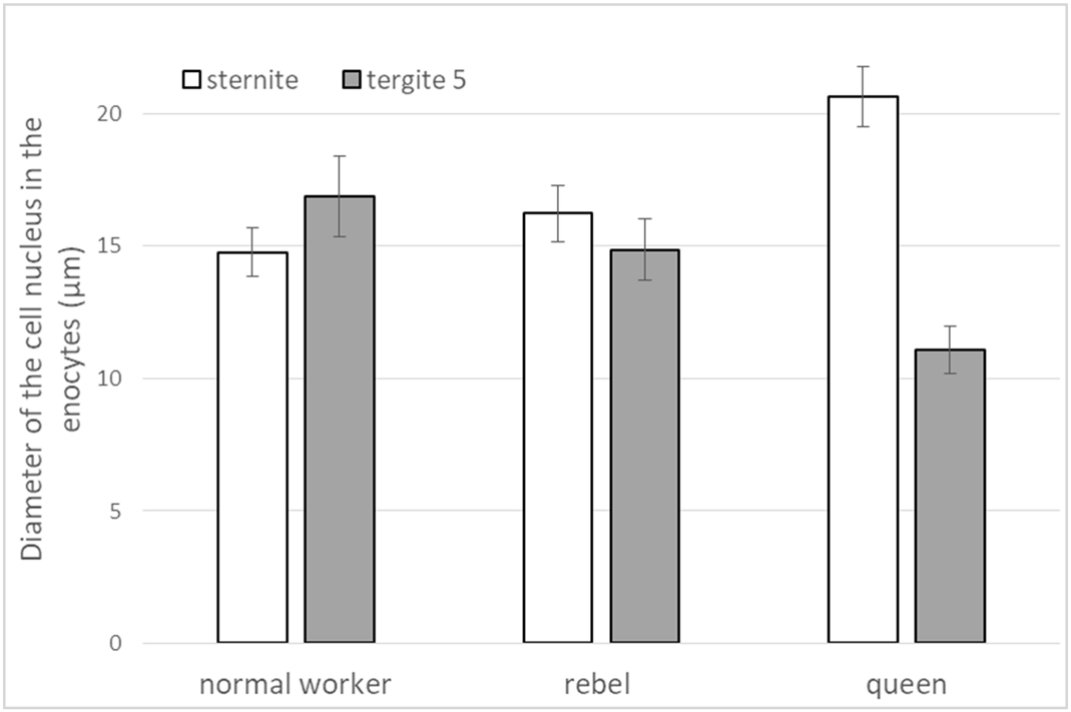
Diameter of the cell nucleus in the oenocytes (µm; mean ± SD) of 1-day-old queens, rebels and normal workers. The differences are statistically significant between averages for rebels and normal workers and queens (Two-way ANOVA with multiple comparison testing using the post hoc Tukey HSD; caste/sub-caste F_2,31_ = 0.45, p = 0.64; tissue F_1,31_ = 169.81, p < 0.001; caste/sub-caste* tissue F_2,31_ = 219.77, p < 0.001; no. of queens = 13; no. of rebels = 14; no. of normal workers = 10). The post-hoc Tukey test showed that there are differences between tissues and castes/sub-caste at max p = 0.023. Tergite 5—the fifth tergite.

Microscopic images of the fat bodies were different between castes/sub-caste and depended on the localisation (Fig. 1, Table 1, Supplementary Fig. S2), similarly to the nuclei diameters of the oenocytes (Fig. 2).

**Table 1:** Similarities and differences in the morphology and biochemical characteristics of the fat body between the queens, rebels and normal workers.

Characteristic	Localisation	Castes/sub-caste		
		Normal workers	Rebels	Queens
**Fat body morphology**				
Number and shape of oenocytes	Sternite	Numerous, oval	Numerous, oval	Numerous, oval
	Tergite 3	Absence	Single, spindle-shaped	Single, various shapes
	Tergite 5	Numerous, oval and spindle-shaped	Numerous, various shapes	Numerous, triangular
Diameter of the cell nucleus in the oenocytes	Sternite	+	++	+++
	Tergite 3	n.a.	+++	++
	Tergite 5	+++	++	+
Layout of lipid droples in the trophocytes	Sternite	Edge, close to the cell membrane	Edge, close to the cell membrane	Throughout
	Tergite 3	Edge, close to the cell membrane	Edge, close to the cell membrane	Throughout
	Tergite 5	Edge, close to the cell membrane; single cells—throughout	Edge, close to the cell membrane; single cells—throughout	Throughout
Intercellular spaces	Sternite	Present	Absent	Absent
	Tergite 3	Present	Present	Absent
	Tergite 5	Present	Absent	Absent
**Biochemical parameters of the fat body**				
Protein concentrations	Sternite	+++	++	+
	Tergite 3	+++	++	+
	Tergite 5	+++	++	+
Glucose concentrations	Sternite	+	++	+++
	Tergite 3	+	++	+++
	Tergite 5	+	++	+++
Glycogen concentrations	Sternite	+	++	+++
	Tergite 3	+	++	+++
	Tergite 5	+	++	+++
Triglyceride concentrations	Sternite	+	++	+++
	Tergite 3	+	++	+++
	Tergite 5	+	++	+++

+ low values; ++ intermediate values; +++ high values; n.a.—not applicable; Tergite 3—the third tergite; Tergite 5—the fifth tergite.

#### The sternite

Regardless of the caste/sub-caste, trophocytes and oenocytes were visible in the sternite fat body (Fig. 1). Characteristic of this fat body localisation was a very high number of oenocytes (Supplementary Fig. S3). Trophocytes (Supplementary Table S1) and oenocytes were the largest in the queens. The lipid drops in the trophocytes were distributed over the entire surface of the cells in the queens, and at the margins in rebels and normal workers. Intercellular spaces were visible in the normal workers. In contrast, the cells were tightly attached to each other in the queens and rebels. The oenocytes had centrally located cell nuclei in all the castes. Diameters of their nuclei were the largest in the queens, whereas the rebels had larger diameters of oenocyte nuclei in comparison to the normal workers (Fig. 2).

#### The third tergite

The queens and rebels had trophocytes and oenocytes in the third tergite, but only trophocyte cells were present in the normal workers (Fig. 1). The trophocytes were especially large in the queens in comparison to the other locations (Supplementary Table S1). Numerous lipid droplets filled entire trophocyte cells in the queens and were present in the vicinity of the cell membrane in the normal workers and the rebels. Intercellular spaces were detected between the fat body cells in the normal workers and the rebels, but not in the queens. Single, triangular/multi-shaped oenocytes were observed in the queens, while they were more abundant in the rebels—oval-shaped with a centrally located cell nucleus. Diameters of the cell nuclei in the queens’ oenocytes were smaller (11.3–13.7 µm) than in the rebels (15.5–22.5 µm).

#### The fifth tergite

Trophocytes and oenocytes were tightly adjacent in the queens and rebels (Fig. 1). Large intercellular spaces were detected in the normal workers. The trophocytes in all the bees were completely filled with lipid drops. Triangular oenocytes were observed in the queens and oval ones in the normal workers and the rebels. The nuclei in the oenocytes were centrally located and their diameters were smaller in the rebels and queens than in the normal workers (Fig. 2).

### Biochemical characteristics of the fat body

**Figure 3:**
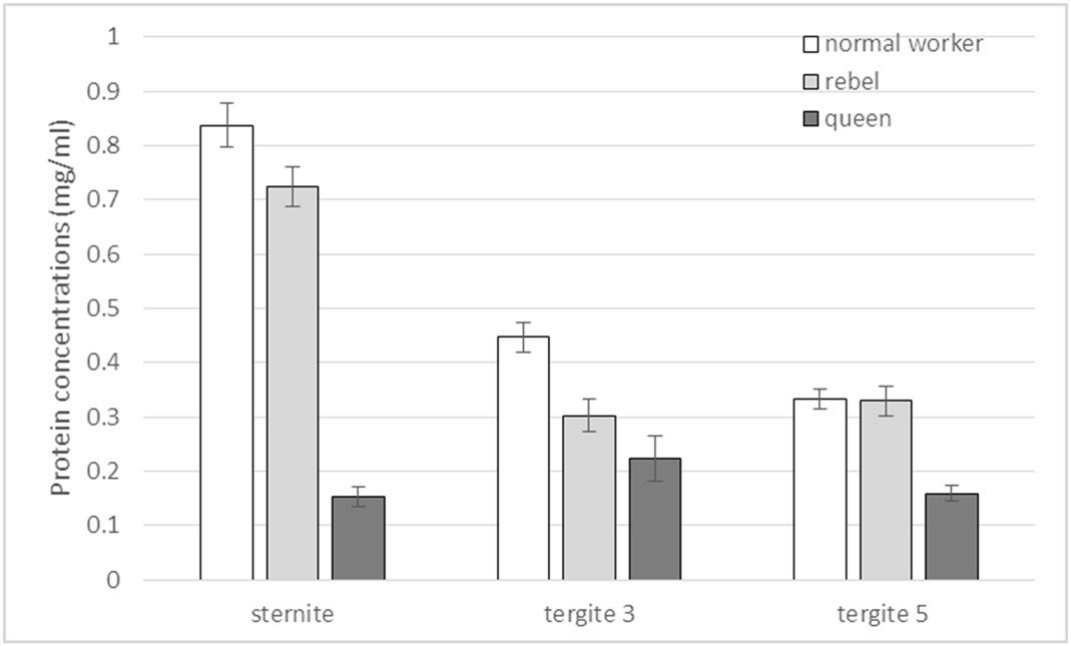
Protein concentrations in the fat body from different localisations in 1-day-old queens, rebels and normal workers. The differences are statistically significant between averages for rebels and normal workers and queens ((Three-Way Anova: colony F_2,513_ = 0.53, p = 0.631; caste/sub-caste F_2,8_ = 1061.73, p < 0.001; tissue F_2,8_ = 3551.10 p < 0.001; colony*caste/sub-caste F_4,513_ = 3.68, p = 0.055; colony*tissue F_4,513_ = 0.79, p = 0.563; caste/sub-caste*tissue F_4,513_ = 938.07, p < 0.001, colony*caste/sub-caste*tissue F_8,513_ = 1.99, p = 0.046) with multiple comparison testing using the post hoc Tukey HSD with p < 0.001 for measurements performed on different castes/sub-castes and tissues. No. of queens = 60; no. of rebels = 60; no. of normal workers = 60). Tergite 3—the third tergite; Tergite 5—the fifth tergite.

**Figure 4:**
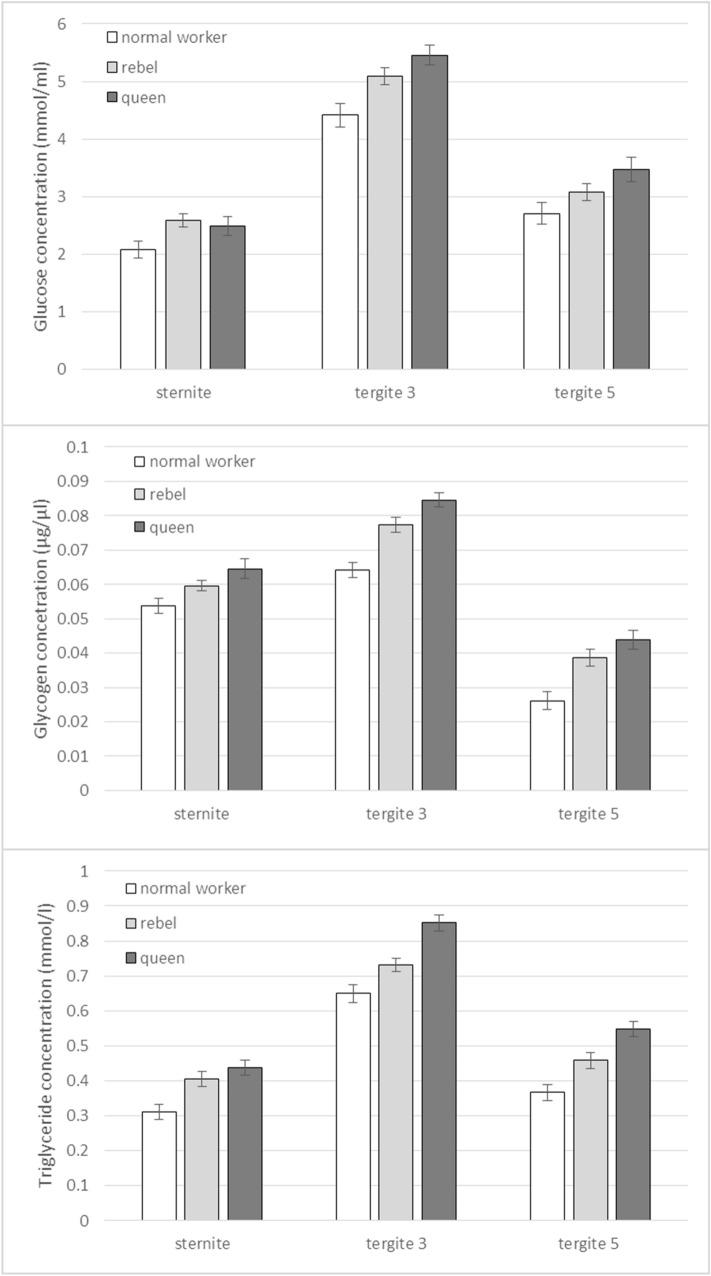
Glucose, glycogen and triglyceride concentrations (mean ± SD) in the fat body from different localisations in 1-day-old queens, rebels and normal workers. The differences are statistically significant between averages for rebels and normal workers and queens ((Three-Way Anova: Glucose: Three-Way Anova: colony F_2,513_ = 7.9, p < 0.001; caste/sub-caste F_2,8_ = 939.2, p < 0.001; tissues F_2,8_ = 11,753.8, p < 0.001; colony*castes/sub-caste F_4,513_ = 2.9, p = 0.023; colony*tissues F_4,513_ = 1.5, p = 0.211; castes/sub-caste*tissues F_4,513_ = 72.0, p < 0.001, colony*castes/sub-caste*tissues F_8,513_ = 1.2, p = 0.299; Glycogen: Three-Way Anova: colony F_2,513_ = 1.4, p = 0.249; castes/sub-caste F_2,8_ = 2247.9, p < 0.001; tissues F_2,8_ = 12,644.8, p < 0.001; colony*castes/sub-caste F_4,513_ = 1.8, p = 0.124; colony*tissues F_4,513_ = 2.0, p = 0.096; castes/sub-caste*tissues F_4,513_ = 78.2, p < 0.001, colony*castes/sub-caste*tissues F_8,513_ = 0.9, p = 0.552; Triglyceride: Three-Way Anova: colony F_2,513_ = 0.9 p = 0.422; castes/sub-caste F_2,8_ = 2718.5p < 0.001; tissues F_2,8_ = 13,445.5, p < 0.001; colony*caste/sub-caste F_4,513_ = 2.6, p = 0.035; colony*tissues F_4,513_ = 1.3, p = 0.272; castes/sub-caste*tissues F_4,513_ = 70.3, p < 0.001, colony*castes/sub-caste*tissues F_8,513_ = 0.8, p = 0.568) with multiple comparison testing using the post hoc Tukey HSD with p < 0.001 for measurements performed on different castes/sub-caste and tissues; no. of queens = 60; no. of rebels = 60; no. of normal workers = 60). Tergite 3—the third tergite; Tergite 5—the fifth tergite.

The concentrations of biochemical parameters were different and depended on the castes/sub-caste and fat body localisation (Figs. 3 and 4). Protein concentrations were the highest in the sternite while concentrations of glucose, glycogen, and triglyceride were the highest in the third tergite, in all the bees under analysis. Protein concentrations were the lowest in the queens (Fig. 3), but concentrations of the remaining compounds were always the highest in the queens (Fig. 4). Analogical concentrations in the rebels assumed values in between those identified for the queens and the normal workers. The fat body masses were the highest in the queens whereas the rebels had higher fat body masses than the normal workers.

The high protein concentrations in the sternite correlated with the numbers of oenocytes (Figs. 1 and 3, Supplementary Fig. S3), whereas the high concentrations of glucose, glycogen, and triglyceride in the third tergite corresponded with the size (diameter) of trophocytes, especially in the queens (Figs. 1 and 4; Supplementary Table S1).

## Discussion

**Figure 5:**
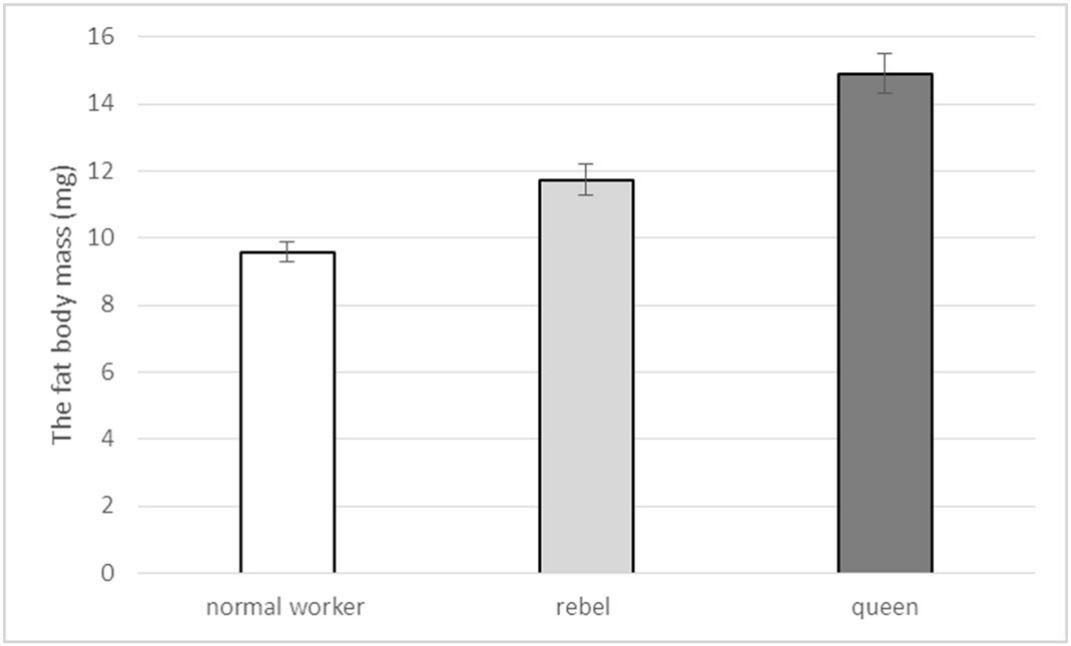
The fat body mass (mg; mean ± SD) of 1-day-old queens, rebel and normal workers. The differences are statistically significant between averages for rebels and normal workers and queens (Two-way ANOVA with multiple comparison testing using the post hoc Tukey HSD test; colony F_2,171_ = 2.3, p = 0.22; castes/sub-caste F_2,4_ = 4378.6, p < 0.001, post-hoc between each groups: p < 0.001; colony*castes/sub-caste F_4,171_ = 0.4, p = 0.787; no. of queens = 60; no. of rebels = 60; no. of normal workers = 60).

Division of labor in social insect colonies derives from conserved pathways regulating reproduction^21,49,50^. Werner et al.^6^ identified a large set (~ 1500) of genes with shared caste-biased abdominal expression in honeybees, with many genes participating in reproduction, such as the *vitellogenin* receptor^51^ and *ovo*^52^. These caste-biased genes are derived from ancient plastically expressed genes (from the distant insect related to *Drosophila *sp.) underlying female reproduction. Through their expression, many of these genes enable the maintenance of homeostasis and proper metabolism in the fat body of bees. Models of the evolution of hierarchical developmental gene-regulatory networks show a relatively small number of highly conserved genes initiate gene cascades, e.g. to set up body patterning, while batteries of downstream genes are evolutionarily labile and largely responsible for lineage-specific/caste features^6,53^. Most likely, this developed during the evolution of the fat body, as the pattern is similar in all the castes and, at the same time, each of the castes has unique features in its different abdominal segments, characteristic only of itself (Figs. 1, 2, 3, 4, 5, Supplementary Fig. S2). We showed that the fat bodies were pleomorphic and also diverse in terms of fat body location (Fig. 1, Supplementary Fig. S2) and functions, as well as the biochemical and physiological processes (Figs. 3, 4, 5) occurring within them. When describing regionalization of the fat body, Haunerland and Shirk^54^, as well as Jensen and Børgesen^55^ focused on the general location of this tissue, e.g. in the head, near the muscles, near the digestive tract. Our results are the first to present the segmentation of the subcuticular fat body, i.e. its differentiation depending on the abdominal segment in which it is present. To emphasize that this segmentation of the fat body applies to all tergites, we have also presented the most characteristic photos for the fourth, sixth and seventh tergites (Supplementary Fig. S2). Although Lu et al.^56^ believe that subcuticular fat body is formed by a single layer of cells, we observed a multi-layer coating of cells around each abdominal segment. These cells adhered tightly to each other in the queens—in the sternite and the third and fifth tergite; while in the rebels—in the sternite and the fifth tergite. In contrast, the intercellular spaces were visible in each segment of the fat body in the normal workers, and the cells were not so tightly attached to each other (Fig. 1). Most likely, the hemolymph is poured into these spaces and there is an exchange of compounds between these tissues^33^. The increased exchange of these compounds, especially in normal workers, maybe the result of an accelerated metabolism that results in a shorter life in comparison to the queens and rebels^28^. In addition, due to the presence of intercellular spaces, the exchange surface of various compounds, which are necessary primarily during the flight and should quickly reach the appropriate tissues (e.g. muscles)^28,57^, increases in workers. This is most likely one of the evolutionary adaptations of workers to their hive-internal versus hive-external tasks.

Hsieh and Hsu^58^ suggested that trophocytes (adipocytes) are large and irregularly shaped, and oenocytes are small and spherical. Our research confirmed this finding, but only in the case of trophocytes and always the largest ones observed in the queens, regardless of the location of the fat body. It is worth emphasizing here that the trophocyte diameters in the third tergite were even as large as 270 µm (Supplementary Table S1), which indicates that they are one of the largest insect trophocytes^42^. In our opinion, it is no coincidence that the largest trophocytes were observed in this tergite. Firstly, it is in close proximity to the heart, ostia, and body cavities. Secondly, according to Paes de Oliveira and Cruz-Landim^37^, it is in the trophocytes that vitellogenin is synthesized, the concentration of which is most probably the highest in the third tergite from where it is immediately directed to the appropriate tissues by the circulatory system. This solution enables a rapid distribution of these substances and ensures the queenְ’s reproductive dominance^59^. Oenocytes had different sizes (Fig. 1, Supplementary Fig. S2). They were large and oval in the sternite fat body, while they were triangular or fusiform or elliptical in the fifth tergite. Their activities were measured according to the diameters of the cell nuclei. It is worth noting that oenocytes located in the fat body in the third tergite were found only in the rebels and queens, i.e. the females with an increased reproductive potential (Fig. 1). Diameters of oenocyte nuclei were larger in the rebels (15.5–22.5 µm) than in the queens (11.3–13.7 µm). Oenocytes are responsible for the synthesis of hormones^33^. Their presence in the reproductive females may indicate an adaptation to the performance of the reproductive function in comparison with sterile females. Thus, the lack of these cells in the third tergite in normal workers (Fig. 1) only supports this thesis. High concentrations of glucose, glycogen, and triglycerides in the queens and rebels, especially in the third tergite (Fig. 4), were most likely the effect of the activities of oenocytes, which metabolically interact with trophocytes, enabling females with an increased reproductive potential to synthesize and maintain energy reserves at a high level^28,60,61^. Triglycerides are the major component of the lipid droplets in trophocytes, and occupy, along with glycogen and protein granules, most of the intracellular space^31,33,34^, especially in the queens (Fig. 1). On the other hand, in the normal workers and the rebels, these droplets were only found under the cell membrane (Fig. 1). It is worth noting that, when pursuing our research, we analysed young virgin queens preparing for the mating flight during which they need the energy reserves^62^. Moreover, during this flight, the queen secretes pheromones whose composition is regulated by lipids^63,64^. Therefore, it can be assumed that the above-mentioned features in maternal trophocytes and also oenocytes are an example of the queen’s adaptations to her reproductive role. Moreover, the highest accumulation of energy reserves in queens (Fig. 4) may be the result of a diet applied during the development, as suggested by Alaux et al.^65^ and Pernal and Currie^66^. However, the question arises: what kind of food is administered to the larvae of the rebels? We already know that they have a shorter ontogenesis as compared to normal workers^67^. If they are fed royal jelly, royalactin activated p70 S6 kinase increases the activity of mitogen-activated protein kinase which is involved in the decreased developmental time, and increases the titer of juvenile hormone, an essential hormone for ovary development^68^. Maintaining the proper relationships between JH, insulin/insulin-like growth factor signaling (IIS) and vitellogenin production in the fat body cells is essential to support egg production^55,69^ and maybe that is why rebels are able to lay eggs^20^. In addition to being glycolytic fuels, glucose and glycogen, present at high levels in queens (Fig. 4), are also used for the synthesis of chitin, a major cuticle component^61,70,71^. Therefore, the queen's cuticle is thicker compared to normal worker bees^72^. The opposite trend was observed for protein as the normal workers had higher protein concentrations in comparison to the females with an increased reproductive potential (Fig. 3). According to Vincent and Wegst^73^, the protein synthetised in the fat body is responsible for the elasticity of the cuticle. The physicochemical properties of cuticle are especially valuable for workers during flights and activities performed in the nest^74^. Lower protein concentrations in the fat body from the sternite and the third and fifth tergite of queens may be the result of constant feeding with royal jelly. However, the reduced protein concentration in rebels may result from low-protein diet since rebels avoid risky tasks, display a delayed onset of foraging behaviour and a stronger tendency to collect nectar compared with normal workers^75^.

The biochemical parameters, in a similar way to the fat body mass, inform about the condition, vitality and longevity, and the immune response of bees^28,76,77^. Highly reproductively potent rebels and queens also had higher fat body masses than normal workers (Fig. 5). Belaid et al.^74^ suggest that young bees have larger fat body masses than old ones. We analysed young bees in our research. The difference in the fat body mass between the reproductive and non-reproductive castes may be due to the maternal need for an increased source of energy for the mating flight. This corresponds with results obtained by Alaux et al.^65^, Brütsch et al.^77^ and Koubová et al.^78^ who claim that a varied, multi-ingredient diet, particularly richer in proteins, increases the fat body mass, especially in queens.

## Conclusions

We confirmed that fat body cells in the queens, rebels and normal workers contain the same components (e.g. nuclei, vacuoles, lipid droplets, and protein and lipid stores), but have original morphological and physiological traits characteristic only of the specific castes, shaped mainly by their reproductive potential (in the evolutionary process). The segmental character of the fat body, which so far has not been considered in scientific research, in the individual insects of a given caste/sub-caste allows for a broader look at the processes taking place in each of those locations. A good example is the presence of oenocytes in the fat body in the third tergite in the females with an increased reproductive capability. Their activities, expressed in the diameter of cell nuclei, correspond with high concentrations of compounds responsible for energy reserves, which are necessary for young queens to perform mating flights. Very large trophocytes in queens, especially in the third tergite of the fat body, are most likely responsible for the synthesis and storage of vitellogenin, which affects the reproductive dominance of these females. The presence of intercellular spaces in workers increases surface for the exchange of various compounds necessary above all during the flight (e.g. in muscles). In summary, the fat body functions as a multitasking system made up of many organs/segments. Each segment works separately, and they all contribute to a common metabolism. Different diameters of oenocyte nuclei and trophocyte sizes, as well as different levels of biochemical parameters indicate the rotational action of individual segments and the activation of cascade biochemical processes. The fat body can thus be compared to the liver, pancreas, spleen and adipose tissues in vertebrates. The rebels, that are more queen-like than normal workers, combine many features of the queens and normal workers in their fat bodies. Our findings can help better understand the ways that led to the origin of different castes in the females of eusocial Hymenoptera and the formation and functioning of the fat body in each of the castes. Considering the above, we suggest that analyzing the fat body in insects and particularly in honeybees, as presented in this paper protocol, should be pursued to enable the (undistorted) visualisation of living tissues.

## Methods

This study was performed at the apiary of the University of Life Sciences in Lublin, Poland (51.224039N–22.634649E). We used four colonies of *Apis m. carnica* honeybees; three of them—the source colonies—were used to obtain larvae of known ages to rear normal workers and rebels; and one (colony 4) for rearing queens.

### Experimental design

A queen was taken from each of the three unrelated source colonies, each of which populated two-box hives (Dadant Blatt; 20 frames; 435 × 150 mm), and caged within a queen-excluder comb-cage containing two empty combs (C1 and C2) for egg laying for 24 h. The third day after the end of egg laying, 50 1-day-old (12–24-h-old) larvae from C1 and C2 were grafted into queen cell cups suspended vertically in the colony no. 4, according to Büchler et al.’s^79^ method. After the larvae were grafted, C1 and C2 were restored to their source colonies with the remaining larvae. Next, each of the source colonies was divided in two equal parts; each in a separate box according to Woyciechowski and Kuszewska’s^17^ procedure. The first part (top box), containing the queen, workers, brood and C1, were used for rearing normal (non-rebel) workers, whereas the other part (bottom box), without a queen but with workers, brood and C2, served for rearing rebels. After sealing the larval cells in C1 and C2, the two boxes were put together again, respectively, so as to restore each of the three source colonies. After 15 days from the moment the eggs were laid, sealed queen cells were placed in an incubator (temperature 34.5 °C, relative humidity 60%). After 18 days, the brood combs C1 and C2 were also placed in this incubator. Twenty bees from each of these combs were captured for morphological and biochemical analyses, as well as twenty additional bees for the determination of the fat body mass.

### Morphological and biochemical analyses

The fat bodies from sternites (between the second and the fifth), the third and the fifth tergites (see^80^; Supplementary Fig. S4) in each of the 60 queens, 60 rebels and 60 normal workers were dissected under a Stereo Zoom Microscope. Each of the fat bodies was dissected and divided in half. The first half was used to take images and measurements of oenocyte nuclear diameters, and the other half for biochemical analyses. The fat bodies from the first half were placed on glass slides in 0.6% natrium chloratum (*pro inj*.) and covered with cover-glasses. Microscopic preparations were observed and the fat body cells were photographed with Camera Olympus DP72 (Microscope Olympus BX61; magnification × 40) with the DIC attachment. This method enables (undistorted) visualisation of living tissues according to Strachecka et al.^81^. The fat bodies from the second half were collected in sterile Eppendorf tubes, containing 25 μl of ice-cooled 0.6% NaCl. Next, the tissues were homogenised at 4 °C and centrifuged for 1 min at 3000*g*. The supernatants were immediately refrigerated at − 40 °C for further biochemical analyses.

The following parameters were determined in the supernatants:

- protein concentrations, using the Lowry method, as modified by Schacterle and Pollack^82^;
- concentrations of triglycerides and glucose, with the colorimetric method using monotests from Cormay (Lublin, Poland) according to the manufacturer’s instructions;
- glycogen concentrations were measured using a Glycogen Assay Kit (K646-100, BioVision).

### Fat body quantification

The fat body mass was estimated using an ether extraction method according to Wilson-Rich et al.^83^. Both queen and worker abdomens were severed from thoraces and dried for 3 days at room temperature. Then, the abdomens were weighed and washed in diethyl ether for 24 h to dissolve the fat. Finally, the abdomens were dried for 3 days and weighed again. The fat body mass was calculated as the difference in abdomen weight after washing with diethyl ether^84^.

### Examination of anatomical characteristics

In order to confirm whether the emerging bees were rebels or normal workers, as well as the queen status, Woyciechowski and Kuszewska’s^17^ method was used to determine the number of ovarioles (ovarian tubules). The total number of ovarioles in both ovaries of each individual was counted. All ovarioles were stained with the Giemsa reagent for approximately 10 s before being measured.

### Statistical analysis

The results were analyzed statistically using Statistica formulas, version 13.3 (2017) for Windows—StatSoft Inc., USA. To compare the number of ovarioles and morphological and biochemical parameters between the rebels and normal workers and queens, a mixed-model two-way and three-way ANOVA was used. The experimental group was a fixed effect, and the colony was a random effect. If a difference among the groups was statistically significant, the ANOVA procedure was followed with multiple comparison testing using the post hoc Tukey HSD test with P = 0.05 as the level of significance.

## Supplementary Information

Supplementary Information.
