# Supplementary material for: Segmentation of the subcuticular fat body in Apis mellifera females with different reproductive potentials
Source: Sci Rep. 2021 Jul 6;11:13887. doi: 10.1038/s41598-021-93357-8 (PMC8260796; doi:10.1038/s41598-021-93357-8)
Supplement: Supplementary file 1 — Supplementary Information. [file 41598_2021_93357_MOESM1_ESM.docx]

**Supplementary material**

**Segmentation of the subcuticular fat body in *Apis mellifera* females with different reproductive potentials**

Aneta Strachecka^1^*, Krzysztof Olszewski^2^, Karolina Kuszewska^3^, Jacek Chobotow^4^, Łukasz Wójcik^1^, Jerzy Paleolog^1^, Michał Woyciechowski^3^


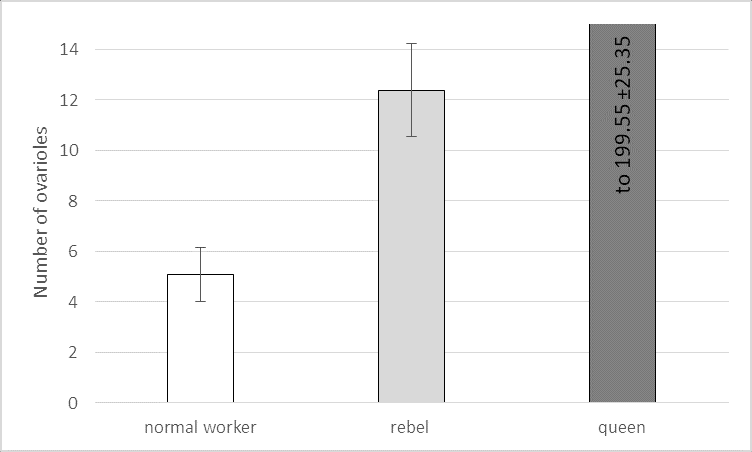


**S1 Fig. Number of ovarioles (mean ± SD) in 1-day-old queens, rebels and normal workers.**

The differences are statistically significant between averages for the rebels and normal workers and the queens (Two-way ANOVA with multiple comparison testing using the post hoc Tukey HSD test; colony F_2, 171_=1.00, p=0.44; castes/sub-caste F_2,4_=1442.48; p=0.000, post-hoc for each group max. p=0.01; colony*castes/sub-caste F_4,171_=2.46, p=0.047, post-hoc for each group max. p=0.02; no. of queens = 60; no. of rebels = 60; no. of normal workers = 60).


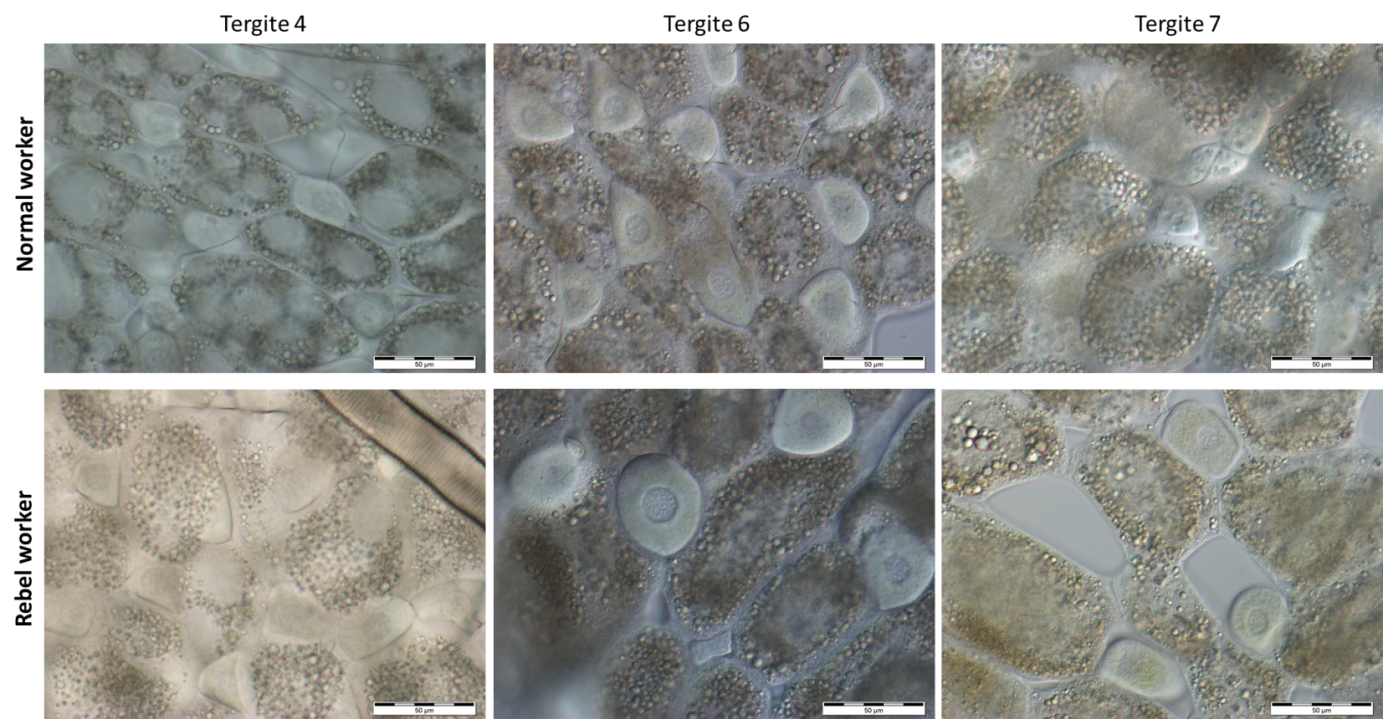


**S2 Fig. Histological features of fat body cells in *Apis mellifera* normal workers and rebels.**

The fat bodies were analysed in the fourth (Tergite 4), sixth (Tergite 6) and seventh (Tergite 7) tergite. Scale bar – 50μm.

**
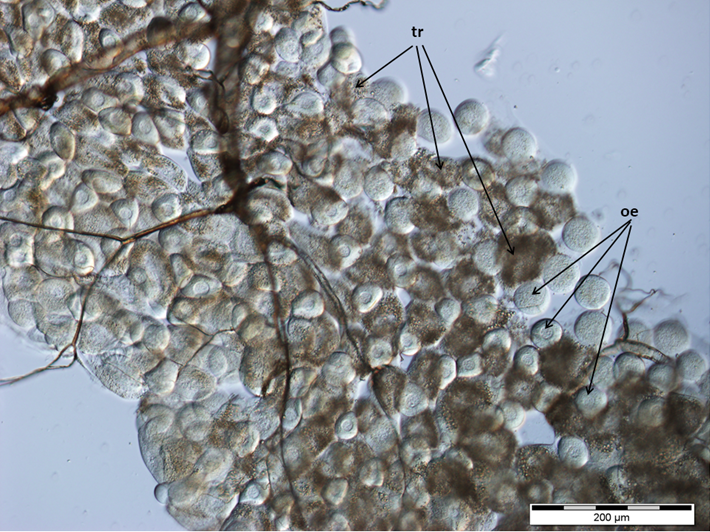
**

**S3 Fig. Histological features of fat body cells from the sternite of the *Apis mellifera* normal workers.**

Scale bar – 200μm. tr – trophocytes; oe – oenocytes.


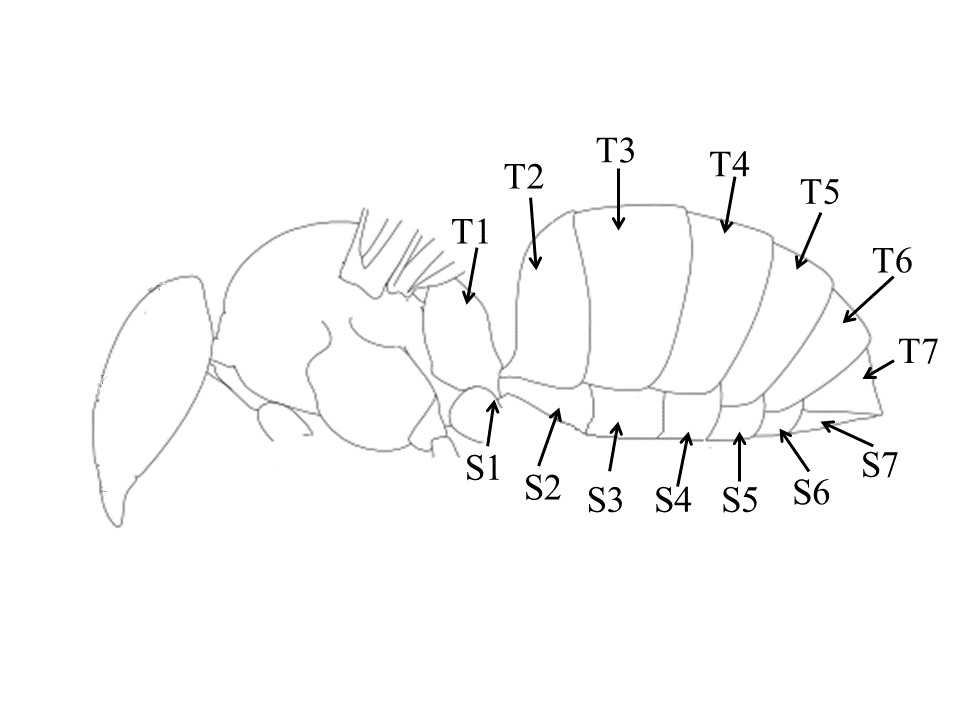


**S4 Fig. The tergite-sternite structure of honey bee abdomen.**

T – tergite; S – sternite; digits 1-7 - numbering of individual segments.

**S1 Table. The ranges of trophocyte diameters (µm) in the respective females and in different locations of the fat body.**

|  | **Sternite** | **Tergite 3** | **Tergite 5** |
| --- | --- | --- | --- |
| Queen | 70-150 | 90-270 | 90-200 |
| Rebel | 40-90 | 30-120 | 40-150 |
| Normal worker | 40-120 | 70-170 | 50-170 |
